# Efficient Recognition and Closed Reduction of Locked Lateral Patella Dislocation

**DOI:** 10.7759/cureus.33415

**Published:** 2023-01-05

**Authors:** Justin Aflatooni, Scott D McKay

**Affiliations:** 1 Department of Orthopedics and Sports Medicine, Houston Methodist Hospital, Houston, USA; 2 Department of Orthopedic Surgery, Texas Children's Hospital, Houston, USA; 3 Department of Orthopedic Surgery, Baylor College of Medicine, Houston, USA

**Keywords:** locked patella dislocation, patella fracture, atraumatic, irreducible closed reduction, irreducible, patella dislocation

## Abstract

Patellar dislocation is not an uncommon injury in the general population that can often be managed in the emergency room with a closed reduction. However, rarely, the patella can become impacted on the lateral femoral condyle and become resistant to closed reduction techniques, which is known as a locked patellar dislocation. This injury is reported in the literature as requiring advanced imaging with a costly workup, operative intervention, or extensive manipulation under general anesthesia, and, to our knowledge, has never been documented to be closed reduced outside the operating room. In this report, we present a 17-year-old male with a locked lateral patella dislocation and describe a new approach to efficiently diagnose and close reduce this injury in the ER under conscious sedation without advanced pre-treatment studies or urgent treatment in the operating room (OR).

## Introduction

Patellar dislocation is not an uncommon injury in the general population, with incidence reported as high as 1:1000, though the higher incidence has been observed in certain populations with predisposing factors (i.e., prior patellar instability events, miserable malalignment syndrome, dysplastic lateral femoral condyle, vastus medialis, etc.) [1]. Given the regional anatomy, the reduction of this injury is rarely inhibited by interposed bone, soft tissues, or other obstacles. However, there have been unique cases of irreducible (otherwise termed "locked") patellar dislocation, both in adult patients and pediatric patients with congenitally abnormal patellar anatomy, that require a reduction in the operating room (OR). In the following case report, we detail a unique case of an adolescent patient with an atraumatic locked lateral patellar dislocation in the absence of congenitally aberrant patellar anatomy that was closed and reduced through a previously undescribed reduction maneuver for patellar dislocation.

## Case presentation

**Figure 1:**
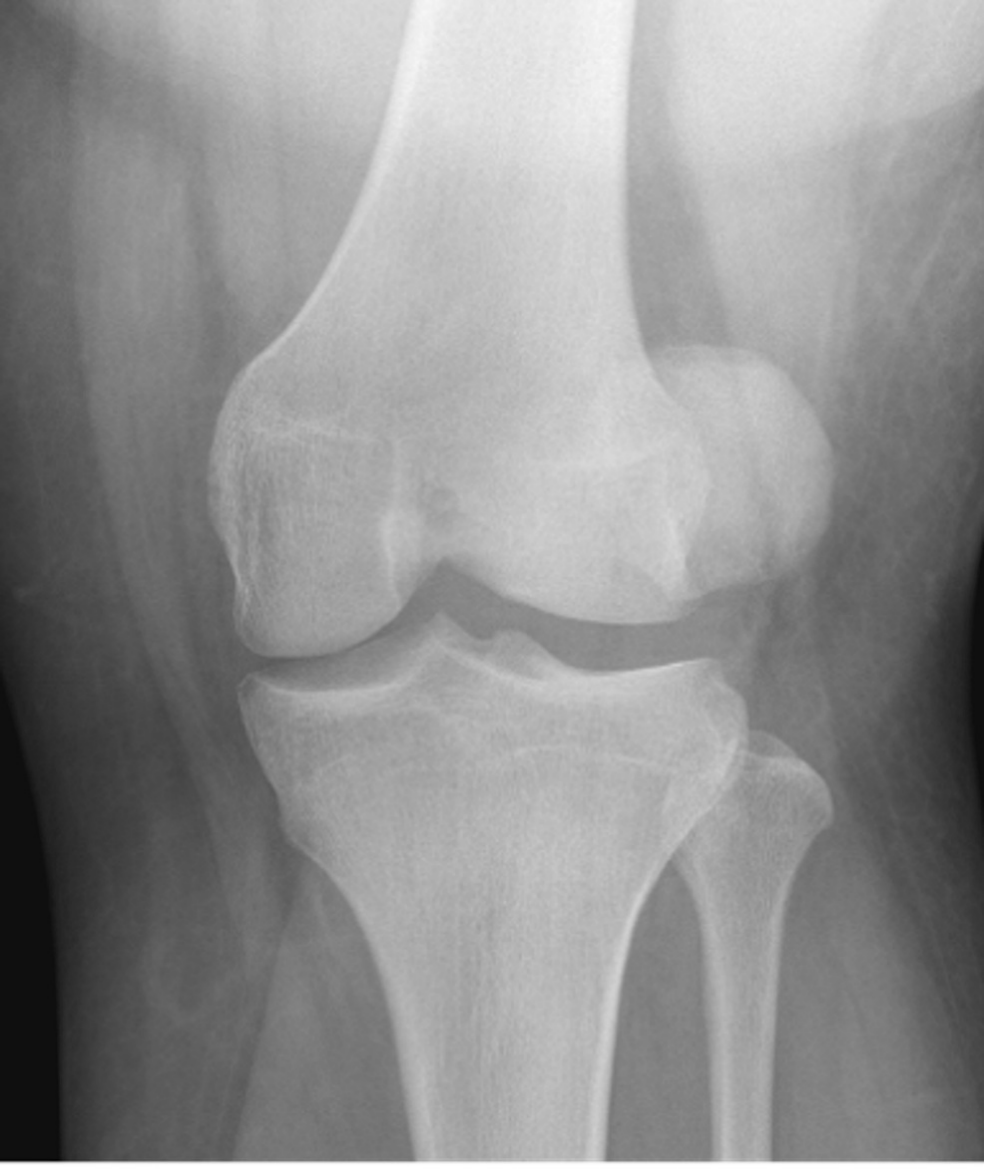
Anterior-posterior radiograph of the left knee showing a lateral patellar dislocation

**Figure 2:**
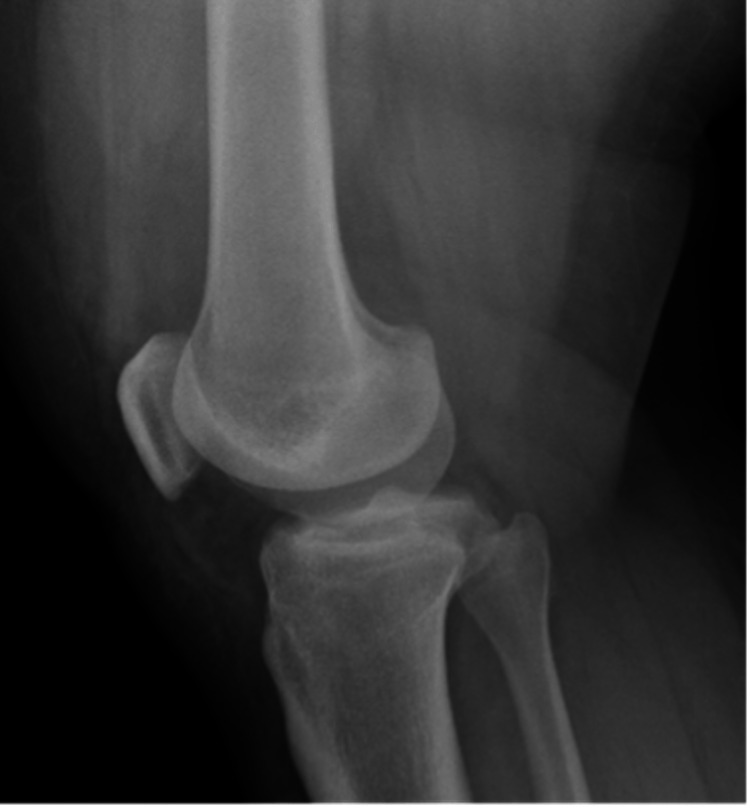
Lateral radiograph of the left knee showing a patellar dislocation

A 17-year-old male with a remote past medical history of three prior right patellar dislocations presented to our emergency department (ED) with a first-time locked left lateral patellar dislocation after pivoting on his left foot during a football game. He underwent a failed attempt at a reduction at the time of the injury by the on-field attending orthopedic physician. The patient was transported to the ED in an ambulance due to the inability to transport him off the field because of his high BMI (body mass index). En route to the ED, he was given nebulized ketamine and temporarily splinted in situ by emergency medical services. In the ED, the splint was removed, radiographs were obtained, and the patient underwent two further failed closed reduction attempts by the ED physician after being given IV fentanyl and nebulized ketamine. At this time, orthopedics was consulted for further management. On examination, the patient had an immobile left knee fixed at 10 degrees of flexion, was neurovascularly intact with no other injuries, and had a patella that remained rigidly fixed in a dislocated position with gentle manipulation. His height and weight were 165 cm and 140.6 kg, respectively (a BMI of 51.6). Radiographs demonstrated a laterally displaced left patellar dislocation (Figures 1, 2).

**Figure 3:**
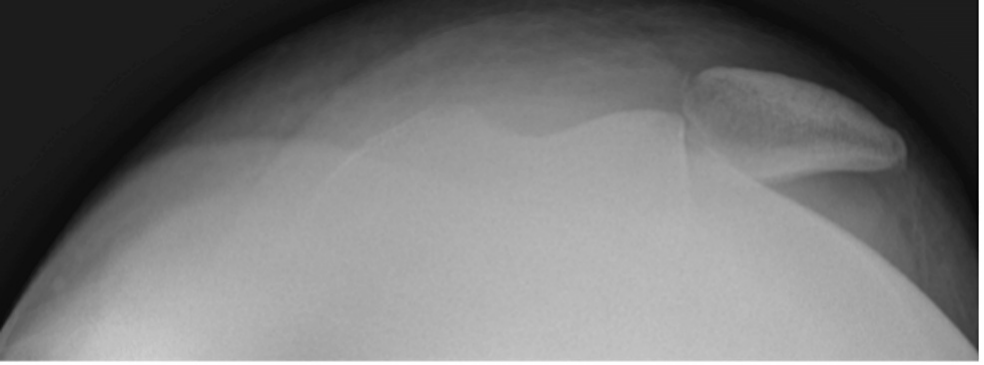
Modified sunrise (patient unable to bend the knee) radiograph of the left knee showing lateral patellar dislocation with medial patellar impaction defect and avulsion fracture of the medial patella

**Figure 4:**
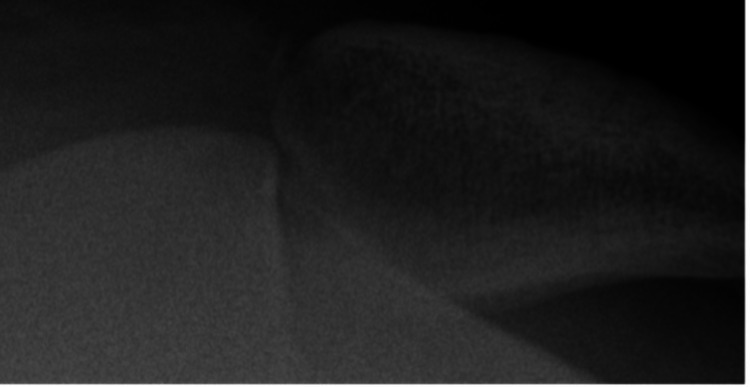
Close-up modified sunrise (patient unable to bend the knee) radiograph of the left knee showing lateral patellar dislocation with medial patellar impaction defect and avulsion fracture of the medial patella

**Figure 5:**
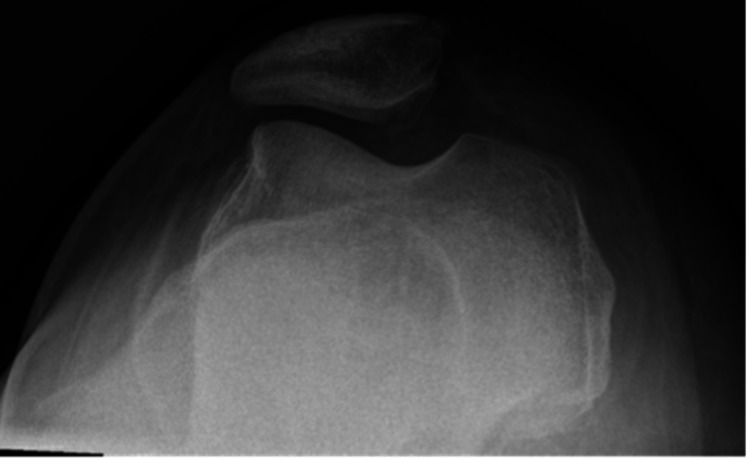
Comparison sunrise radiograph of the right knee showing laterally subluxated patella without congenital anatomic abnormality that may predispose the injured side to locked dislocation

At this time, a modified sunrise view of the left patella and a sunrise view of the right patella (for comparison) were obtained. Careful evaluation of the sunrise views demonstrated a medial avulsion fracture of the patella with hinging of the patella on the lateral trochlear ridge via an acute impacted medial patellar defect (Figures 3-5).

**Figure 6:**
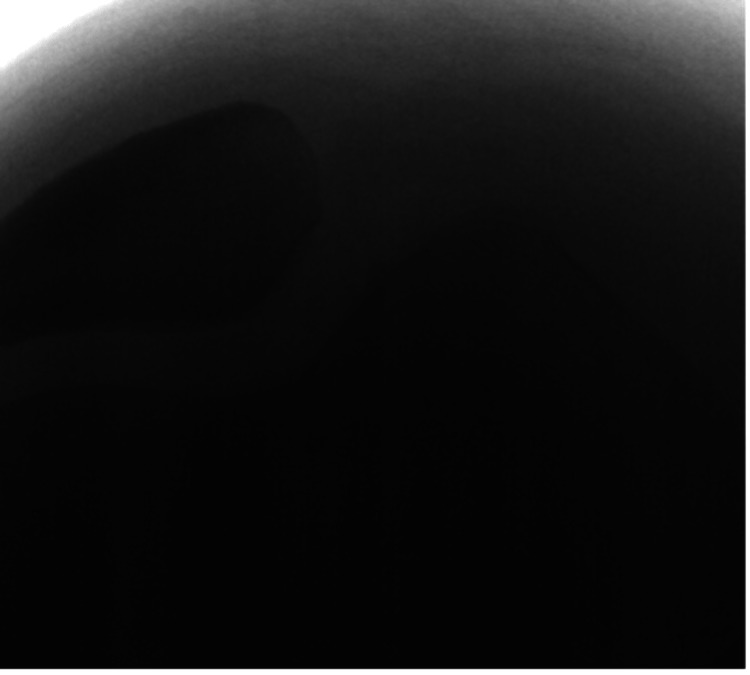
Intra-reduction fluoroscopy sunrise radiograph of the left knee confirmed a successfully reduced patella

The patient was then consciously sedated with IV ketamine to assure adequate relaxation of the musculature and a further closed reduction attempt in the ED. Under conscious sedation, the patella remained rigidly fixed against the initial anteromedial relocation force with the knee fully extended. This maneuver would have normally reduced a typical patella dislocation but was unsuccessful. Therefore, this novel technique was employed: with the application of a firm superolateral force with the knee extended, the patella readily unlocked, and a subsequent superomedial force reduced the injury. Subsequent post-reduction exam under sedation was improved and displayed a ligamentously stable knee that had regained full mobility; although it displayed lateral tilt, it was found to track within the trochlea with knee flexion under sedation and was confirmed on the intra-sedation sunrise view with mini-c-arm fluoroscopy (Figure 6).

**Figure 7:**
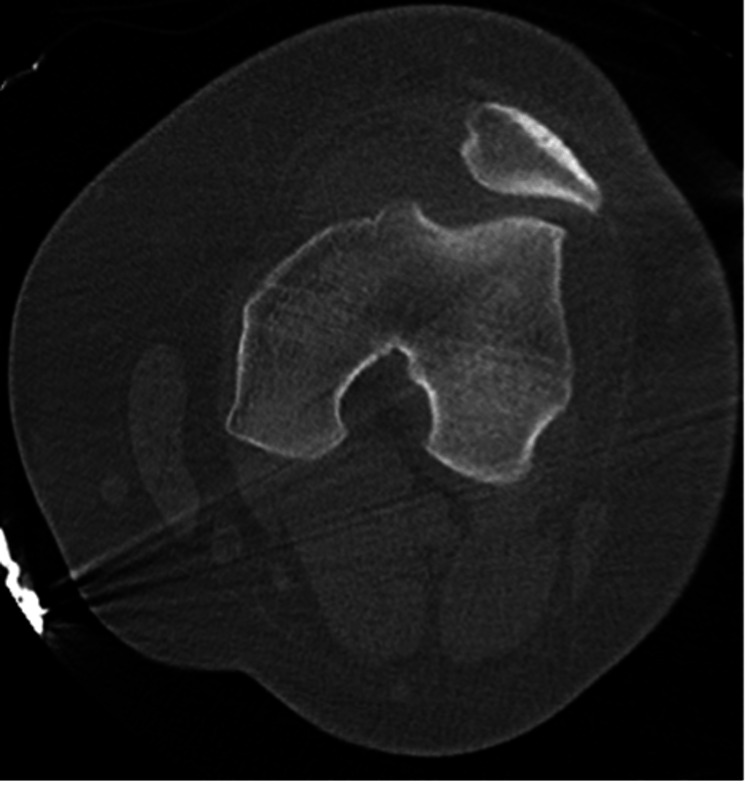
Post-reduction axial computed tomography image emphasizing the acute impaction defect of the left medial patella

The patient was placed in a hinged knee brace locked in extension. Subsequent post-reduction treatment included computed tomography (patient or guardian declined acute MRI for soft tissue evaluation, due to ER wait times), which confirmed reduction, the lack of unrecognized fractures or intra-articular osseous bodies, the lack of previously unrecognized soft tissue injury, and better characterized the previously appreciated medial avulsion fracture and impaction defect of the patella (Figure 7).

Based on patient and family preference, no other acute treatments were conducted, and he was discharged to follow-up in the clinic for further treatment; clinic follow-up had not occurred at the time of manuscript submission, and therefore concomitant acute soft tissue injuries are currently not known.

## Discussion

In this case, a healthy 17-year-old male athlete sustained an atraumatic lateral patellar dislocation that was resistant to normal reduction maneuvers secondary to an acute medial impaction on the lateral trochlear ridge. In this case, the patella became mobile and amenable to reduction only after accentuating the dislocation.

To our knowledge, this is the first time this injury has been described in the literature in an adolescent patient. Arguably, more importantly, this is the first description in the literature of an efficient reduction maneuver for this injury that did not require closed reduction under general anesthesia in the operating room or open reduction. Tanos et al. describe a similar injury in a middle-aged patient who had an irreducible lateral patella dislocation that was impacted on the lateral trochlea and went on to need a reduction in the operating room with intra-articular lateral pressure via arthroscopic means [2]. They concluded that arthroscopic management of this rare injury is an effective reduction method prior to open treatment in adult patients [2]. Our patient underwent a successful, previously undescribed, closed reduction maneuver in the ED under conscious sedation with ketamine. In our case, this saved the patient admission, a prolonged stay in the ED and hospital, and a costly trip to the operating room.

Delagrammaticas et al. report on another adult patient with an atraumatic irreducible lateral patellar dislocation impacted on the lateral trochlea with similar imaging characteristics to our patient, though in their case a larger medial patellar fracture was present that did not block reduction [3]. In their case, they took their patient to the operating room for open reduction and concluded that high suspicion is necessary to recognize this type of injury with early advanced imaging and open treatment to avoid numerous unsuccessful closed reduction attempts [3]. In our patient, once orthopedics was consulted, careful analysis of the sunrise radiographs combined with reports of previous unsuccessful reduction attempts and a careful physical exam revealing a rigidly fixed patella was enough to recognize the unique pathology and form a successful reduction treatment. Similarly, Yerimah et al. report on an adult patient with a traumatic lateral patellar dislocation that was not reducible in the ED and went on to require open surgical treatment [4]. Their intra-operative findings included a patella with a medial impaction defect like the one we found on our pre-reduction radiographs and better characterized on our post-reduction CT. Based on their intra-operative findings and manipulation, they attribute the locked patella to the lateral trochlea "keying-in" to the impaction defect on the medial patella [4]. Grewel et al. report on a locked traumatic lateral patellar dislocation that failed multiple closed reduction attempts in the ED under conscious sedation and was ultimately reduced in the OR with closed reduction [5]. In their study, they describe reduction after 20 minutes of significant anterolateral force to the patella and cite an incarcerated fat pad as a proposed block to their reduction [5]. However, given the amount of force and time necessary, we believe it is more likely secondary to a bone defect, as previously described in other articles. Furthermore, in their study, they found eight reports of lateral femoral condyle impaction causing locked lateral patella dislocation dating back to 1941, only two of which were under 18 years of age, and all of which were treated with open treatment [5].

Previous case reports of this injury type in adult patients have demonstrated concomitant osteochondral defects with or without chondral loose bodies, which were treated with either open or arthroscopic management [2-4]. Given the likely mechanism of this injury, these injuries likely create significant chondral damage to the patella. Though they may ultimately go on to require surgery, depending on the associated pathology, if reduction can be achieved, management of concomitant injuries may be effectively done in the outpatient setting.

Altogether, we feel this study contributes to the literature as the first described locked lateral patella dislocation in an adolescent patient that was treated via a closed means with a novel reduction maneuver using clinical assessment and pre-reduction radiographs to make the diagnosis. In the absence of large fractures or lesions that may obviously block reduction, an acutely locked lateral patellar dislocation may be the result of impaction on the trochlear border and necessitate "unlocking" the patella through further lateral dislocation prior to normal reduction maneuvers. In our case, careful clinical assessment of the history of the present illness with a physical exam and anterior/posterior, lateral, and bilateral sunrise views alone may allow for an efficient closed reduction in the ED without the need for more costly or invasive interventions in the acute setting.

## Conclusions

A careful clinical assessment of the history of the present illness, a physical exam, and radiographs are sufficient for a proper diagnosis of this injury. This injury can be closed and reduced using the described technique, without needing general anesthesia or operative intervention, in the acute setting. Though soft tissue injuries may co-exist that warrant future operative intervention, in the acute setting, this injury may be treated like conventional patella dislocations and followed up in the clinic for further treatment after an acceptable reduction.
